# Metabolomic and Biochemical Analysis of Two Potato (*Solanum tuberosum* L.) Cultivars Exposed to In Vitro Osmotic and Salt Stresses

**DOI:** 10.3390/plants10010098

**Published:** 2021-01-06

**Authors:** Bahget Talat Hamooh, Farooq Abdul Sattar, Gordon Wellman, Magdi Ali Ahmed Mousa

**Affiliations:** 1 Department of Arid Land Agriculture, Faculty of Meteorology, Environment and Arid Land Agriculture, King Abdulaziz University, Jeddah 21589, Saudi Arabia; bhamooh@kau.edu.sa; 2 Division of Biological and Environmental Sciences and Engineering, King Abdullah University of Science and Technology, Thuwal 23955, Saudi Arabia; gordon.wellman@kaust.edu.sa; 3 Department of Vegetables, Faculty of Agriculture, Assiut University, Assiut 71526, Egypt

**Keywords:** drought, salinity, phenols, trehalose, antioxidant, micropropagation, screening

## Abstract

Globally, many crop production areas are threatened by drought and salinity. Potato (*Solanum tuberosum* L.) is susceptible to these challenging environmental conditions. In this study, an in vitro approach was employed to compare the tolerance of potato cultivars ‘BARI-401’ (red skin) and ‘Spunta’ (yellow skin). To simulate ionic and osmotic stress, MS media was supplemented with lithium chloride (LiCl 20 mM) and mannitol (150 mM). GC-MS and spectrophotometry techniques were used to determine metabolite accumulation. Other biochemical properties, such as total phenols concentration (TPC), total flavonoids concentration (TFC), antioxidant capacity (DPPH free radical scavenging capacity), polyphenol oxidase (PPO), and peroxidase (POD) activities, were also measured. The two cultivars respond differently to ionic and osmotic stress treatments, with Spunta accumulating more defensive metabolites in response, indicating a higher level of tolerance. While further investigation of the physiological and biochemical responses of these varieties to drought and salinity is required, the approach taken in this paper provides useful information prior to open field evaluation.

## 1. Introduction

Potato (*Solanum tuberosum* L.) is the leading tuber crop [1], with beneficial nutritional impact [2,3], but is very sensitive in terms of food security [4,5]. Potato is grown in relatively cool climates and is sensitive to drought [6] due to its shallow root system [7], moderately tolerant to salinity, although highly sensitive during tubers formation [8,9]. Globally, desertification and salinization are currently affecting about 10% of arable land, which decrease yield more than 50% in major crops [10,11]. Salinity and drought interrupt many physiological and biochemical processes, causing osmotic and oxidative stress, ion imbalance, mineral deficiency, and ion toxicity problems [12]. Hence, understanding of plant tolerance to drought and salinity is a critical research topic [13].

One of the major signatures of abiotic stress leading to yield reduction is the overproduction of reactive oxygen species (ROS) [14]. These ROS include superoxide anions (O_2_•^−^), hydrogen peroxide (H_2_O_2_), singlet oxygen (^1^O_2_), and hydroxyl radicals (OH•), which can be damaging and cause different physiological, molecular, and biochemical responses [15]. ROS are also vital signaling molecules that alert plants to adjust their metabolism [16] to adapt to adverse environments. Additionally, production of secondary metabolites like phenols, flavonoids, tannins, saponins, and anthocyanin have been reported in different plant species under abiotic stresses [17]. These alterations may be positive or negative depending on many factors, such as plant species, developmental stage, and stress duration [18].

In response to stress, plants adopt defense strategies such as: ion homeostasis, activation of antioxidant enzymes, and production of different organic compatible solutes [19,20]. Plants tolerant to abiotic stresses can be identified by applying selective agents such as NaCl (for salinity), PEG, or mannitol (for drought) [19]. Lithium (Li), an analogue of sodium, has been used elsewhere for studying ionic toxicity under saline conditions [21]. Successful in vitro selection for drought and salt tolerance using different selection agents has been applied to crops, including potato [12]. As a low tolerance to salinity and drought are major limitations to potato cultivation, development of stress-tolerant varieties is important to enhance crop productivity [19].

Metabolomics is one of the developing “*omics*” techniques critical to the study of abiotic stress tolerance of crops [22]. It aims to decode, by metabolite determination, the probable effect of abiotic stresses on all dynamic biochemical processes of plant cells [23]. Metabolomics techniques such as gas chromatography-mass spectrometry (GC-MS) are useful to identify, assess, and evaluate the response of important metabolites [24]. In plant abiotic stress research, an important aim is to examine particular metabolites that are critical in tolerance and metabolic variations leading to different responses [25,26]. Different metabolomics approaches have been used to investigate salinity stress in crops, including barley, tomato, maize, and wheat [27,28,29]. Similarly, for drought tolerance, metabolites profiling of various crops such as soybean, barley, wheat, and rice, have been recorded [30,31,32,33,34].

Plant tissue culture approaches can be applied to pinpoint characteristics, and to simplify abiotic stresses experiments in a controlled environment. In vitro techniques can also be used to activate genes already present in plant genome via epigenetic changes [35]. The study presented here screened two potato cultivars: ‘BARI-401’ (red skin) and ‘Spunta’ (yellow skin), for osmotic and ionic stress responses, by in vitro culture using Murashege and Skooge (MS) medium [36], supplemented with 20 mM LiCl and 150 mM mannitol. These experiments may help to identify genotypes with improved ionic and osmotic tolerance less expensively and more time effectively than open field trials [37]. Hence, this work aims to explore the performance of these cultivars by using different selective agents for ionic (LiCl) and osmotic (mannitol) stress response assessed by metabolomics finger printing and biochemical analysis.

## 2. Results

### 2.1. Biochemical Analysis under Different Levels of LiCl and Mannitol

**Figure 1:**
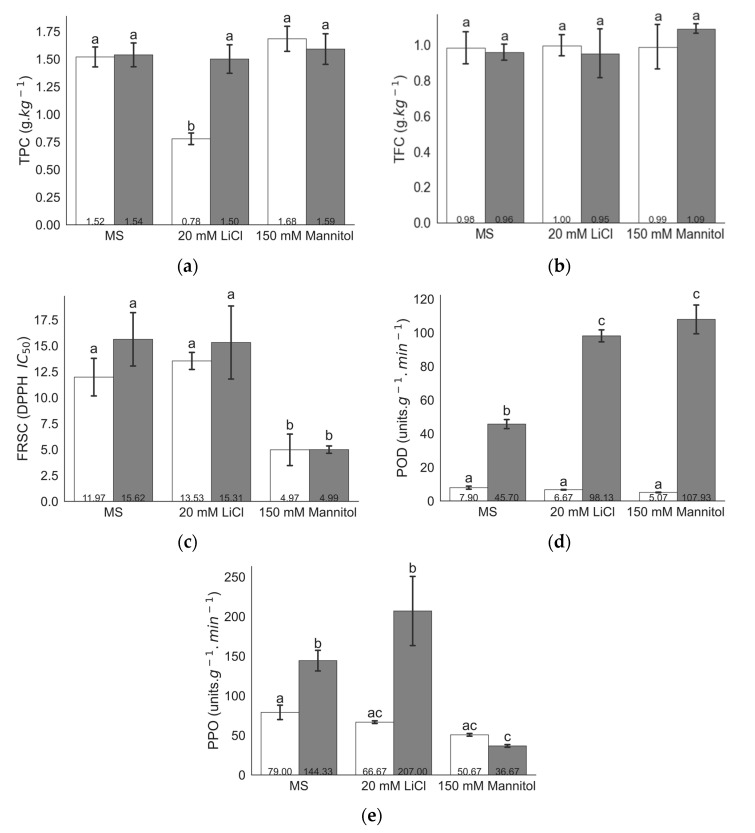
Biochemical analysis of cultivars BARI-401 (white) and Spunta (grey) grown on MS with addition of 20 mM LiCl or 150 mM mannitol. (**a**) Total phenols content (TPC), (**b**) total flavonoid content (TFC), (**c**) free radical scavenging capacity (FRSC DPPH IC_50_ value), (**d**) polyphenol oxidase (PPO), and (**e**) peroxidase (POD) activity. Values are means ± standard deviation (SD) (*n* = 3) with letters indicating significant differences between cultivars and treatments, determined by two-way analysis of variance (ANOVA) and Tukey-Kramer HSD (*p* ≤ 0.05).

BARI-401 showed lower (*p* ≤ 0.05) total phenols concentration (TPC) in LiCl treatment compared to control grown plantlet, while TPC was similar to control under mannitol treatment (Figure 1a). The treatments had no significant effect on total flavonoids concentration (TFC) (Figure 1b). Antioxidant capacity measured by DPPH free radical scavenging capacity (FRSC) was higher (lower DPPH, half-maximal inhibitory concentration (IC_50_) value) in mannitol treatment compared to control and LiCl (Figure 1c). Similarly, activity of antioxidant enzymes POD and PPO activity were lower in mannitol compared to control and LiCl treatment (Figure 1d,e). In Spunta, TPC and TFC were not affected at tested LiCl and mannitol concentrations (Figure 1a,b), however antioxidant capacity measured by DPPH free radical scavenging capacity was higher (lower DPPH IC_50_ value) in mannitol treatment compared to control and LiCl (Figure 1c). Plantlets treated with either 20 mM LiCl or 150 mM mannitol had increased (*p* < 0.01) POD activity compared to control and all BARI-401 samples. In 150 mM mannitol, plantlets had reduced (*p* < 0.01) PPO activity compared with controls (Figure 1d,e).

### 2.2. Changes in Metabolite Profile Induced by LiCl and Mannitol Exposure

**Figure 2:**
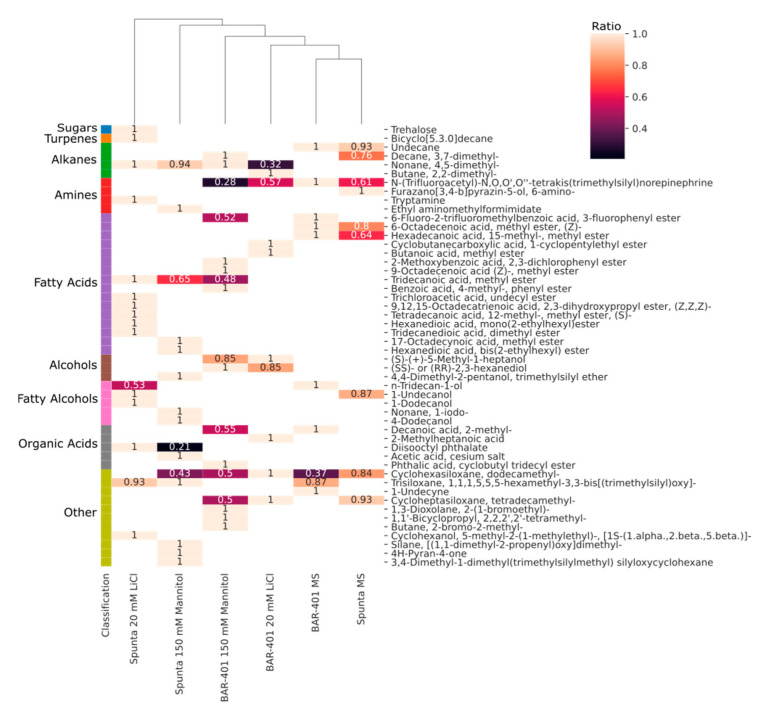
Heat map and cluster hierarchical analysis of 50 metabolites recorded from methanolic shoots extract of BARI-401 and Spunta cultivated on MS media, with addition of 20 mM LiCl and 150 mM Mannitol. Where detected, compounds are annotated and show color variation according to ratio of signal intensity to the max intensity of each compound amongst treatments.

**Figure 3:**
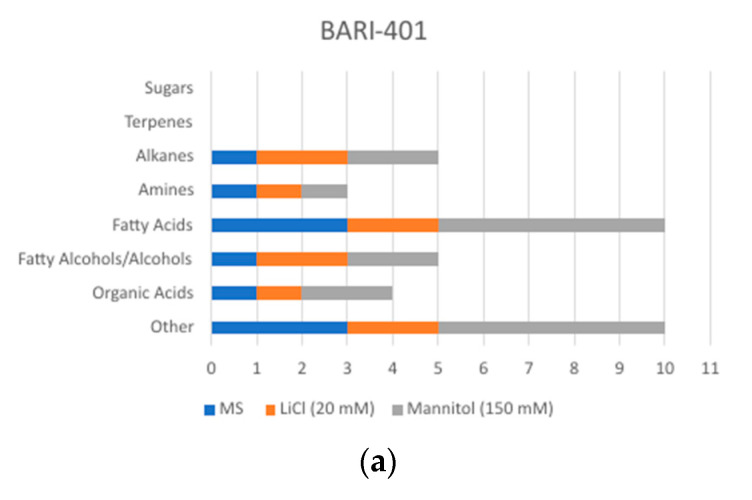

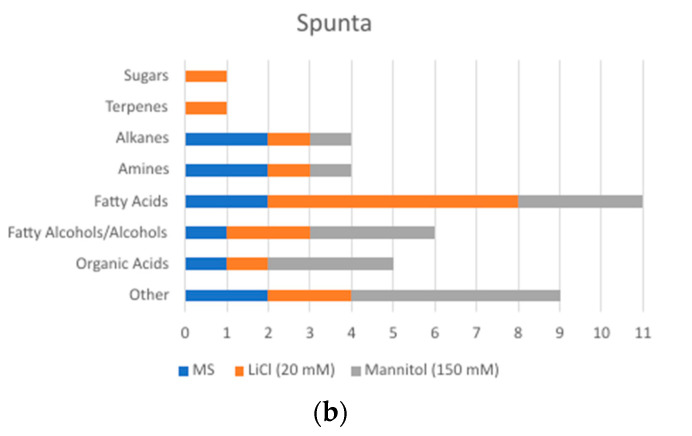
Distribution of different chemical groups detected by GC-MS in (**a**) BARI-401 and (**b**) Spunta cultivated on MS medium (MS) and MS with addition of 20 mM LiCl and 150 mM mannitol.

A total of 50 different compounds were identified by GC-MS in methanolic shoots extracts of both varieties (Figure 2) and were classified into 9 groups (Figure 3a,b). Both cultivars exhibited considerable changes in detected compounds and signal intensity in LiCl and mannitol treatments (Supplementary Table S5: Metabolites detected in shoots).

**Figure 4:**
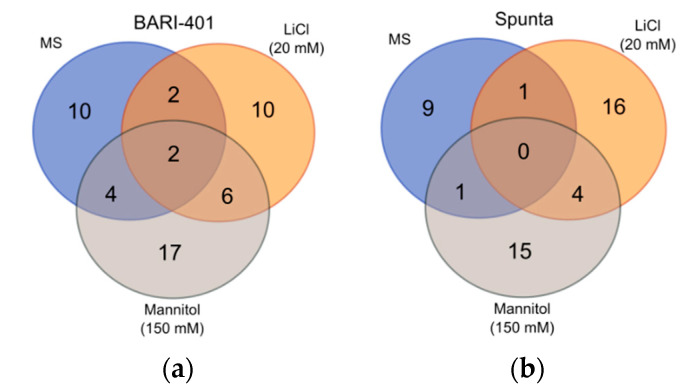
Venn diagram showing number of metabolites produced by BARI-401 (**a**) and Spunta (**b**), cultivated on MS medium (MS) and MS with addition of 20 mM LiCl and 150 mM mannitol.

In BARI-401, a total of 30 unique compounds were identified in treated plantlets (10 control, 10 LiCl, and 17 mannitol). LiCl-treated plantlets shared two compounds with control while mannitol shared four compounds with control. Only six compounds were found common between LiCl and mannitol treatments and only two compounds were found in all treatments (Figure 4a). The distribution of different groups of compounds varied with organic acids the highest in mannitol-treated plants, and fatty alcohols/alcohols were higher in mannitol and LiCl treatments than control. Fatty acids were most abundant in mannitol treatment, followed by control and LiCl treatments. Different alkanes (two in LiCl, two in mannitol treatments, and one in control) were also detected, with the amine norepinephrine detected in all the treatments (Figure 4a, and Supplementary Table S5).

In Spunta, a total of 36 unique compounds were identified (9 control, 16 LiCl, 15 mannitol). LiCl- and mannitol-treated plantlets shared only one compound each with control. Only four compounds were found common between LiCl and mannitol treatments and no compounds were shared by all treatments (Figure 4b). Similar to BARI-401, the mannitol treatment had the highest number of organic acids detected, followed by LiCl. Fatty alcohols/alcohols were also higher in mannitol treatment. Among the treatments, LiCl treatment produced more fatty acids than mannitol and control treatments, respectively. Interestingly, the only sugar, Trehalose, was found in LiCl treatment, along with a terpene, Bicyclo[5.3.0]decane. Alkanes were also found in all treatments (Figure 3b and Supplementary Table S5).

### 2.3. Principal Component Analysis (PCA) of Treatment Variables

**Figure 5:**
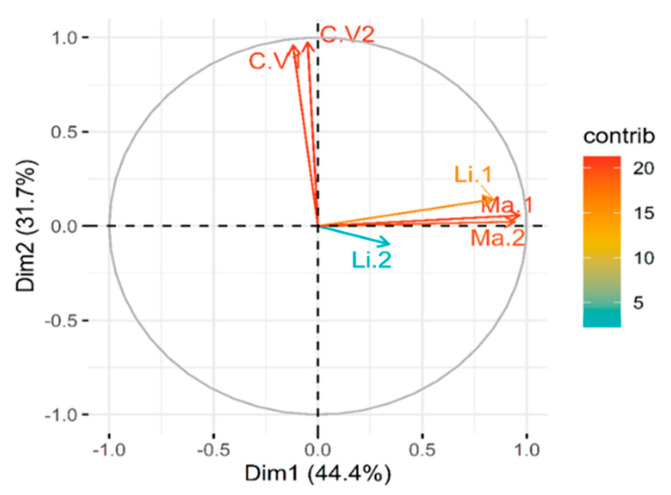
Principal component analysis (PCA) for C.V1 (Control BARI-401), C.V2 (Control Spunta), Li.1,2 (LiCl 20 mM BARI-401 and Spunta) and Ma.1,2 (Mannitol 150 mM BARI-401 and Spunta). The circle has a correlation value of 1.0, and arrow lengths are proportional to the correlation coefficient for each variable.

A total of 66% of variance was explained by the two principal components (Dimensions, PC) of six treatment variables: 20 mM LiCl (BARI-401 Li.1 and Spunta Li.2), 150 mM Mannitol (BARI-401 Ma.1 and Spunta Ma.2), and 2 controls (BARI-401 C.V1 and Spunta C.V2) (Figure 5). Four treatment variables: Li.1, Ma.1, Li.2, and Ma.2, are correlated positively with one another. As shown in Figure 5, the arrows of these variables are clustered together, indicating positive correlation. However, these four variables were independent from C.V1 and C.V2.

## 3. Discussion

Screening of in vitro cultures of plant tissues with different selective agents like PEG, mannitol, or sorbitol for drought [19] and NaCl or LiCl for salinity [38] stresses can help to identify plants with desirable tolerance characteristics and to investigate metabolic variations that are induced by abiotic stresses [26], such as the production of reactive oxygen species (ROS) [14], organic solutes [20], and flavonoids [39]. This approach has been previously employed to increase tolerance by selecting explants that survive under these conditions [40].

In the present study, the two cultivars examined respond differently to LiCl stress, with BARI-401 having reduced TPC compared to control treatment, in contrast to Spunta which does not vary (Figure 1a). Antioxidant phenolic compounds play a role as scavengers of ROS in plants [41] and can help provide resistance against both biotic and abiotic stresses [42,43]. Previous studies have observed that high concentration of salts in media have an adverse effect on the concentration of phenolic compounds in two wild relatives of potato, *S. stoloniferum* and *S. bulbosum* [39], and in *Capsicum annuum* [44]. In these wild potato species, salinity stress also did not significantly increase flavonoids concentrations [39], similar to our results. Rousses [45] also observed that total phenolic compounds, flavanols, and polyamines concentrations in Jojoba (*Simmondsia chinensis*) explants subjected to 50–500 mM mannitol were reduced with increasing levels of stress, possibly due to reduced activity of flavonoids biosynthetic enzymes [13].

The two cultivars also show differences in PPO and POD activity (Figure 1d,e). PPO activity has been proposed to be induced under mild drought stress and declines under high drought stress, as seen in *Aeluropus lagopoides* [46] and in sugar beet [47]. Increase in PPO activity may also result in degradation of accumulated phenolic compounds [47].

In our experiments, the PPO activity of Spunta is significantly higher in control and LiCl treatments compared to that of BARI-401 and is decreased only under mannitol treatment, while the PPO activity of BARI-401 under both treatments is similar to control levels (Figure 1d). There does not appear to be a correlation between PPO activity and changes in TPC accumulation (Figure 1a). These results suggest that Spunta may be more responsive to stress and potentially more tolerant comparatively.

Despite the differences between PPO and POD activity between the two cultivars, overall, free radical scavenging activity was similar between cultivars, with both BARI-401 and Spunta showing increased FRSC under 150 mM mannitol. Increased antioxidant capacity of both cultivars under mannitol suggests that these cultivars have a similar response to osmotic stress. Variation in non-enzymatic antioxidant capacity has previously been seen in potato [39]. Other experiments on free radical scavenging ability of extracts of germinating grapes seeds under osmotic stress were shown to be weak but suggest that phenolic content and antioxidant capacity are positively correlated [48,49]. Differences in antioxidant activity may related to degree of stomatal closure or other responses that change the rate of CO_2_ fixation [50]. Additionally, this increased free radical scavenging may be contributed by other compounds such as carotenoids [51].

Drought and salinity stress may increase the accumulation of ROS and induce detoxification responses of plants, such as increased production of ROS scavenger enzymes like CAT, POX, POD, APX, PPO, and SOD [52]. It has been observed in cotton that salinity stress induces ROS scavenging enzymes in salt-tolerant cultivars but is unchanged or reduced in non-tolerant cultivars [53,54,55]. Similarly, the role of POD and CAT in ROS detoxification was found to vary in potato cultivars and suggests that the importance of ROS detoxification as a tolerance mechanism is cultivar-dependent [56]. Similarly, Demirel et al. [7] reports relatively unchanged antioxidant enzymes activities in the sensitive potato cultivar ‘Agria’ when exposed to stress.

Plant responses to abiotic stresses involve post-translational changes in proteins leading to modification and accumulation of various metabolites, resulting in specific physiological responses [57]. Osmotic adjustment by accumulation of ions and compatible solutes to combat osmotic effects [58] is a basic mechanism to protect plants under abiotic stress [59].

Although limited, the metabolomics fingerprinting by GC-MS of the two cultivars in this study revealed variations both between cultivars and between LiCl and mannitol treatments (Figure 2), potentially indicating differences in their stress tolerance ability. The metabolites of the six different variables (two varieties by three treatments) submitted to PCA separated along two principal components, showing almost 66% of the experimental variation (Figure 5). This analysis shows that control treatments are negatively correlated to the stress treatments, supporting that variation in metabolites detected between treatments exist. Based on the hierarchical clustering (Figure 2) and examining the variations in signal intensity, Spunta under LiCl and mannitol treatments varies more from both control grown plantlets and BARI-401 under all conditions. Although, without appropriate internal standardization, it is difficult to compare signal intensities, and these results indicate that Spunta responds to stress by inducing more changes in metabolite composition than BARI-401.

Several notable differences in metabolite accumulation between the two cultivars deserve mention. In Spunta, trehalose sugar was detected in 20 mM LiCl treatment. Previously, trehalose has been detected by GC-MS in soil grown potato tubers [60] and in Arabidopsis [61]. Stress-tolerant plants are known to accumulate non-reducing disaccharides like trehalose when exposed to stress [62]. Increased level of trehalose sugar has been recorded in wheat cultivars under salinity and drought [63]. Likewise, increased trehalose has been observed in maize plants’ leaves, cob, and at the silking stage [64], and in rice plants [65] under salinity treatments. Transgenic expression of microbial trehalose biosynthesis genes in tobacco [66,67,68], rice [69,70], tomato [71], and potato [72], can also improve stress tolerance. Trehalose itself is an important osmolyte and osmo-protectant [73,74] and may reduce the permeability of salt by maintaining the integrity of plasma membranes or play a role as an antioxidant [75].

Carbohydrate metabolism is directly associated with photosynthetic activities and plays an important role in stress tolerance. Plants utilize starch and fructans as an energy source in stress conditions rather than glucose [76,77]. Trehalose-6-phosphate (T6P) in plastids regulates photosynthesis and starch production [78,79]. Starch synthesis begins through activation of ADP-gluscose pyrophosphorylase (AGPase) via posttranslational redox modification by thioredoxin dependent on SNF1-related kinase (SnRK1) expression [80] (Supplementary Figure S1). Approximately 1000 genes in Arabidopsis have been shown to respond to SnRK1 control [81]. Consequently, variations in trehalose and T6P concentrations impact many biological functions, including adaptive stress responses [82]. The detection of trehalose in LiCl-treated Spunta plantlets may indicate an adaptive stress response not present in BARI-401.

Our study also indicated an increased presence of saturated fatty acids like myristic acid (Tetradecanoic acid, 12-methyl-, methyl ester, (S)-) and Stearic acid (17-Octadecynoic acid, methyl ester) in LiCl and mannitol treatments, especially in Spunta (Figure 3b). Unsaturated fatty acids composition also increased with α-lenolenic acid (9,12,15-Octadecatrienoic acid, 2,3-dihydroxypropyl ester) produced in Spunta under LiCl treatment. Fatty acids, like suberin and cutin, are important extracellular lipid polymers that safeguard against adverse environmental conditions through reshaping membrane fluidity [83]. Our results are similar to those reported by Khalid et al., in which rice cell cultures adapted to 25 mM LiCl have increased saturated fatty acids levels compared to un-adapted cultures [84]. An increase in unsaturated fatty acids has also been observed in *Suaeda salsa* L. [85], safflower [86], *Brassica olearacea* [87], and Arabidopsis [88] under salt stress. Overexpression of ω-3 desaturases, which increase C18:3 fatty acid composition, also increase salt and drought stress tolerance in tobacco [89]. The inherent level of fatty acids’ unsaturation is important in salt and drought stress tolerance [90]. Elevated concentrations of saturated fatty acids play a role in membrane fluidity reduction, which reduces the flow of ions through the membrane [91]. Conversely, unsaturated fatty acids are negative regulators of membrane fluidity [84]. The capability for adjusting membrane lipid fluidity through altering fatty acids levels is important in acclimation to stress and mainly dependent on activity of fatty acids desaturases [92].

Finally, several alkanes were detected in the two varieties in this study. The wax structure of leaf cuticle is predominated by alkanes (50–70%) [93] and plays an important role in resistance to drought [94]. The changes in alkane biosynthesis indicated by our results may reflect alteration of the wax compositions of leaf cuticles in response to stress, although this needs to be examined further.

The differences in metabolite composition, in particular the presence of saturated fatty acids and trehalose accumulation, suggests that Spunta may have a higher capacity for tolerance to LiCl and mannitol stress than BARI-401. Conversely, the fewer changes in BARI-401 metabolites compared to control grown plants may indicate a higher tolerance as tolerance mechanisms are not yet induced.

## 4. Materials and Methods

### 4.1. In Vitro Potato Plantlets Growth and Treatment with LiCl and Mannitol

Tubers of *Solanum tuberosum* L. cultivars BARI-401 and Spunta were sourced from Astra Food Company Ltd., Tabuk, Saudi Arabia. Plantlets were grown from healthy and homogeneous tuber sprouts. After sterilization with 70% *v*/*v* ethanol and 20% *v*/*v* commercial bleach [35], sprouts were cultured in Duran poly-carbonated tissue culture bottles (Duran^®^ Schott, Germany) containing 50 mL autoclaved (15 min at 121 °C and 15 psi) MS medium [33] with phytagel 4 gL^−1^, sucrose 30 gL^−1^, 6-Benzylaminopurin (BAP) 2 mgL^−1^, Indolebutyric acid (IBA) 1 mgL^−1^, and 0.25 mgL^−1^ Gibberellic acid (GA3) [36]. The pH of the media was adjusted to 5.7–5.8 by using 0.1M HCL.

Stem nodal segments were sub-cultured every 4 weeks to obtain sufficient plantlets for experiments. For stress treatments, randomly selected uniform plantlets of each cultivar were transferred to MS medium (control) and MS with addition of 20 mM LiCl (LiCl treatment) [38] or 150 mM mannitol (mannitol treatment) [95]. Plant growth regulators BAP 2 mgL^−1^, IBA 1 mgL^−1^, and 0.25 mgL^−1^ GA_3_ [96] were supplemented in all media. Plantlets were grown for 45 days before harvest and further analysis.

### 4.2. Sample Preparation, Extraction, and Biochemical Analysis of Potato Plantlets

#### 4.2.1. Extract Preparation for Total Phenols (TPC), Total Flavonoids (TFC), and Antioxidant Activity

Potato fresh shoots (2 g/sample) were selected randomly and extraction was done as per Awad et al. [51]. 20 mL of 80% methanol was mixed with sample and shaken at 150 rpm for 12 h, then was filtered with Whatman^®^ filter paper No. 1 at room temperature.

#### 4.2.2. Methanol Total Phenols Concentration (TPC) Estimation

The TPC was measured following Hoff and Singleton’s [97] protocol. A mixture of 50 µL methanolic extract, 100 µL of Folin-Ciocalteu reagent, and 850 µL of methanol was prepared and kept at 23 + 1 °C for 5 min. Sodium carbonate (20% *w*/*v*) was then added to the mixture and left for 30 min to react. TPC absorbency was measured at 750 nm. Results were expressed in g.kg^−1^ fresh weight (FW) Gallic acid equivalent after quantification from the calibration curve obtained from gallic acid absorbance at known concentrations.

#### 4.2.3. Total Flavonoids Concentration (TFC) Estimation

The TFC was measured by a revised colorimetric method as described by Zhishen et al. [98]. 250 µL of methanolic extract was mixed with 1.25 mL of water and 75 µL of 5% *w*/*v* NaNO_2_. The solution was held for 6 min before mixing with 150 µL 10% *w*/*v* AlCl_3_, 0.5 mL NaOH (1 M), and 275 µL distilled water. Absorbance at 510 nm was recorded for total flavonoids. The calibration curve was obtained from the absorbance of known concentrations of catechin for quantification of total flavonoids and the results were given as g.kg^−1^ FW catechin equivalent.

#### 4.2.4. Antioxidant Capacity by DPPH Free Radical Scavenging Capacity (FRSC) Assay

Methanolic extract of in vitro potato shoots were analyzed for free radical scavenging activity in DPPH (1,1-diphenyl-2-picrylhy-drazyl) methanol [42]. 0.1 mL of methanolic extract and 0.9 mL of fresh DPPH methanol solution (0.1 mM) were mixed. As a control, the same quantity of methanol was used. After dark incubation at room temperature for 30 min, the absorbance was noted at 517 nm. Percent scavenging activity was calculated by the following formula:

DPPH radical scavenging (%) = [(Abs control−Abs sample)/Abs control] × 100

The dose response curves were used to calculate IC_50_ (inhibition concentration) values.

#### 4.2.5. Enzymes Activity Evaluation

To prepare crude enzyme extract, 1 g of shoot samples was homogenized with Tris-HCl (20 mM) buffer (pH 7.2) then centrifuged at 10,000 rpm for 10 min at 4 °C [39]. The supernatant was stored at −20 °C prior to peroxidase (POD) and polyphenol oxidase (PPO) assays.

##### Peroxidase (POD) Assay

Peroxidase (EC 1.11.1.7) activity was examined as described by Mar’ia and Cascone [99] and Awad et al. [51]. The reaction mixture consisted of 1000:10 µL of H_2_O_2_ (0.97 M), 80 µL of guaiacol (0.5 M) respectively, 250 µL of sodium acetate buffer (pH 5.5), and 50 µL crude extract. After one minute to allow for guaiacol oxidation, absorbance at 470 nm was recorded. Per unit activity of enzyme is the quantity of enzyme required for 1.0 O.D. min^−1^ change under standard assay conditions.

##### Polyphenol Oxidase (PPO) Assay

Polyphenol oxidase (EC 1.14.18.1) activity was examined by catechol substrate following the methodology of Jiang et al. [100]. 200 µL of crude extract was mixed with 2800 µL of catechol (20 mM) solution in 0.01 M sodium phosphate buffer (pH 6.8). An absorbance increase at 400 nm was recorded over 3 min. Results are expressed as per unit activity of enzyme required for 0.1 O.D. min_−1_ change under standard assay conditions.

### 4.3. Potato Shoots Sample Preparation for GC-MS Metabolites Analysis

Shoot samples were prepared for metabolite analysis following the method of Roessner et al. [60]. Three independent biological replicates were prepared from each treatment (mannitol, LiCl, and control) for both cultivars (18 samples total). Shoots were harvested, snap-frozen in liquid nitrogen, and stored at −80 °C. 100 mg of frozen sample was ground by mortar and pestle into a fine powder in liquid nitrogen and extracted in 1.4 mL methanol.

Due to constraints, only one biological/technical replicate of each cultivar and treatment combination was examined by GC-MS. Sample methanol extracts (1 µL) were injected with an automatic sampler and separated on a QP2010 plus series GC fixed with a split/splitless injector port and determined with a FID QP 2010 plus mass selective detector. Rtx 5 ms 0.25 mm ID (column) and 0.25 µm film thickness (df) (Shimadzu, Japan) was used to perform GC. The injector temperature was 250 °C and the flow rate of helium carrier gas was at 1 mL.min^−1^. Ion source temperature was 210 °C and interface temperature was kept at 250 °C. Temperature of the oven was at isothermal 60 °C for five minutes followed by a stepwise rise at 5 °C.min^−1^ until the oven temperature touched 300 °C and held for a further minute. Before the next injection, temperature was equilibrated at 60 °C for 6 min. At 3 scans.s^−1^, mass spectra were recorded in the 50–500 m/z range. Peaks were identified by the automated mass spectral deconvolution and identification program (AMDIS) and the National Institute of Standards and Technology (NIST library, version v. 2.0 f).

### 4.4. Experimental Design and Statistical Analysis

For the experiment, a completely randomized design (CRD) was used, with two varieties and three treatments: MS medium (control), MS with 20 mM LiCl, and MS with 150 mM mannitol. Three biological replications were conducted per treatment. The results were analyzed by analysis of variance (ANOVA) using Python (version 3.6.3). The Tukey-Kramer HSD test was used to separate the differences between treatments at probability level *p* ≤ 0.05. Moreover, to examine the relationships between the six different variables, principal component analysis (PCA) was performed by R studio (version 1.2.5033) software. Scripts used in analysis available via Github (https://github.com/gbwellman/2020PotatoGCMSanalysis). Venn diagrams were generated using the online tool provided by Ghent University, Bioinformatics and Evolutionary genomics group (http://bioinformatics.psb.ugent.be/webtools/Venn/).

## 5. Conclusions

This study examined the response of two potato cultivars to ionic and osmotic stress in vitro. The results discussed above indicate that Spunta shows a greater response to ionic and osmotic stress than BARI-401 by increased POD/PPO activity, reduced ROS production, trehalose accumulation, and increased saturated fatty acids composition. The interplay between TPC and free radical scavenging activity in BARI-401 and Spunta under mannitol stress needs further examination.

The GC-MS results identify multiple compounds that may be involved in stress responses or tolerance mechanisms and warrant further study. Further validation, both by in planta testing and growth under field conditions [101], is required to determine the impact of these differences on the abiotic stress tolerance of these two cultivars. The in vitro approach used in this study, with the novel insights provided by GC-MS, may be used as a preliminary trial prior to field evaluation [12,102]. Identification of traits correlated to increased ionic and osmotic tolerance, such as POD/PPO activity or trehalose accumulation, will allow this approach to rapidly screen potato genotypes to assist in selection and breeding of potato cultivars with improved tolerance.

## Data Availability

The data presented in this study are openly available in FigShare at https://doi.org/10.6084/m9.figshare.13518899.

## Supplementary Materials

The following are available online at https://www.mdpi.com/2223-7747/10/1/98/s1, Table S1: Representing different metabolites in BARI-401. Table S2: Representing different metabolites in BARI-401. Table S3: Representing different metabolites in Spunta. Table S4: Representing different metabolites in Spunta. Table S5: Metabolites detected in shoots. Figure S1: Trehalose pathway role in eukaryotes. Trehalose-6-phosphate (T6P) controls sugar metabolism and plant development. Trehalose and glucose are also responsible for many signaling and regulatory pathways and integrate external cues to adapt cells to abiotic stress, growth, and development The diagram is adapted from Reference [103]. Metabolites detected in shoots. Figure S2: GC-MS chromatography intensity readings of cv. BARI-401. Control (top), LiCl 20 mM (middle), Mannitol 150 mM (bottom). Figure S3: GC-MS chromatography absolute intensity readings of cv. Spunta. Control (top), LiCl 20 mM (middle), Mannitol 150 mM (bottom).
