# Supplementary material for: Metabolomic and Biochemical Analysis of Two Potato (Solanum tuberosum L.) Cultivars Exposed to In Vitro Osmotic and Salt Stresses
Source: Plants (Basel). 2021 Jan 6;10(1):98. doi: 10.3390/plants10010098 (PMC7825055; doi:10.3390/plants10010098)
Supplement: Supplementary file 1 [file plants-10-00098-s001.pdf]

Supplementary Tables and Figures

Table S1: Representing different metabolites in 'BARI- 41'

Treatments	Organic/Fatty acids	Alkanes	Amines
Control	6-Fluoro-2-trifluoromethylbenzoic acid, 3-fluorophenyl ester<br>Decanoic acid, 2-methyl-<br>6-Octadecenoic acid, methyl ester, (Z)-<br>Hexadecanoic acid, 15-methyl-, methyl ester	Undecane	norepinephrine
LiCl (20 mM)	2-Methylheptanoic acid<br>Cyclobutanecarboxylic acid, 1-cyclopentylethyl ester<br>Butanoic acid, methyl ester	Nonane, 4,5-dimethyl-<br>Butane, 2,2-dimethyl-	Norepinephrine
Mannitol (150 mM)	6-Fluoro-2-trifluoromethylbenzoic acid, 3-fluorophenyl ester<br>Phthalic acid, cyclobutyl tridecyl ester<br>2-Methoxybenzoic acid, 2,3-dichlorophenyl ester<br>Decanoic acid, 2-methyl-<br>9-Octadecenoic acid (Z)-, methyl ester<br>Tridecanoic acid, methyl ester<br>Benzoic acid, 4-methyl-, phenyl ester	Nonane, 4,5-dimethyl-<br>Decane, 3,7-dimethyl-<br>Butane, 2-bromo-2-methyl-	norepinephrine

Table S2: Representing different metabolites in 'BARI-41'

Treatments	Fatty Alcohol	Miscellaneous
Control	n-Tridecan-1-ol	Cyclohexasiloxane, dodecamethyl-
		Trisiloxane, 1,1,1,5,5,5-hexamethyl-3,3-bis[(trimethylsilyl)oxy]-
		1-Undecyne
LiCl (20 mM)	(S)-(+)-5-Methyl-1-heptanol	Cyclohexasiloxane, dodecamethyl-
	(SS)- or (RR)-2,3-hexanediol	Cycloheptasiloxane, tetradecamethyl-
Mannitol (150 mM)	(S)-(+)-5-Methyl-1-heptanol	Cyclohexasiloxane, dodecamethyl-
	(SS)- or (RR)-2,3-hexanediol	Cycloheptasiloxane, tetradecamethyl-
		1,3-Dioxolane, 2-(1-bromoethyl)-
		1,1'-Bicyclopropyl, 2,2,2',2'-tetramethyl-

Table S3: Representing different metabolites in 'Spunta'

Treatment	Organic/Fatty acids	Amines	Fatty Alcohol
Control	6-Octadecenoic acid, methyl ester, (Z)-	norepinephrine	1-Undecanol
	Hexadecanoic acid, 15-methyl-, methyl ester	Furazano[3,4-b]pyrazin-5-ol, 6-amino-	
LiCl (20 mM)	Trichloroacetic acid, undecyl ester	Tryptamine	N-tridecanol
	Tridecanoic acid, methyl ester		1-Dodecanol
	9,12,15-Octadecatrienoic acid, 2,3-dihydroxypropyl ester, (Z,Z,Z)-		
	Tetradecanoic acid, 12-methyl-, methyl ester, (S)-		
	Hexanedioic acid, mono(2-ethylhexyl)ester	norepinephrine	(S)-(+)-5-Methyl-1-heptanol
	Tridecanedioic acid, dimethyl ester		(SS)- or (RR)-2,3-hexanediol
	Diisooctyl phthalate		

Mannitol (150 mM)	Tridecanoic acid, methyl ester<br>17-Octadecynoic acid, methyl ester<br>Hexanedioic acid, bis(2-ethylhexyl) ester<br>Acetic acid, cesium salt<br>Diisooctyl phthalate	Ethyl aminomethylformimidate	Nonane, 1-iodo-<br>4,4-Dimethyl-2-pentanol, trimethylsilyl ether<br>4-Dodecanol

Table S4: Representing different metabolites in ‘Spunta’

Treatment	Miscellaneous	Sugars	Terpenes
Control	Cyclohexasiloxane, dodecamethyl-<br>Cycloheptasiloxane, tetradecamethyl-<br>Undecane<br>Decane, 3,7-dimethyl-		
LiCl (20 mM)	Trisiloxane, 1,1,1,5,5,5-hexamethyl-3,3-bis[(trimethylsilyl)oxy]-<br>Cyclohexanol, 5-methyl-2-(1-methylethyl)-, [1S-(1.alpha.,2.beta.,5.beta.)]-<br>Nonane, 4,5-dimethyl-	Trehalose	Bicyclo[5.3.0]decane
Mannitol (150 mM)	Cyclohexasiloxane, dodecamethyl-<br>Trisiloxane, 1,1,1,5,5,5-hexamethyl-3,3-bis[(trimethylsilyl)oxy]-<br>Silane, [(1,1-dimethyl-2-propenyl)oxy]dimethyl-<br>4H-Pyran-4-one<br>3,4-Dimethyl-1-dimethyl(trimethylsilylmethyl) silyloxycyclohexane<br>Nonane, 4,5-dimethyl-		

Table S5: Metabolites detected in shoots

Compounds	'BARI-41'			'Spunta'		
	Control	LiCl 20 mM	Mannitol 150 mM	Control	LiCl 20 mM)	Mannitol 150 mM
Undecane	497452	0	0	464319	0	0
N-(Trifluoroacetyl)-N,O,O',O''-tetrakis(trimethylsilyl)norepinephrine	33340	18946	9202	20399	0	0
Cyclohexasiloxane, dodecamethyl-	40951	110517	54819	92309	0	47347
Trisiloxane, 1,1,1,5,5,5-hexamethyl-3,3-bis[(trimethylsilyl)oxy]-	34031	0	0	0	36395	39232
6-Fluoro-2-trifluoromethylbenzoic acid, 3-fluorophenyl ester	9362	0	4828	0	0	0
n-Tridecan-1-ol	168079	0	0	0	89703	0
Decanoic acid, 2-methyl-	76880	0	41994	0	0	0
1-Undecyne	12619	0	0	0	0	0
6-Octadecenoic acid, methyl ester, (Z)-	68646	0	0	54661	0	0
Hexadecanoic acid, 15-methyl-, methyl ester	58901	0	0	37909	0	0
Furazano[3,4-b]pyrazin-5-ol, 6-amino-	0	0	0	6956	0	0
Decane, 3,7-dimethyl-	0	0	49538	37761	0	0
Cycloheptasiloxane, tetradecamethyl-	0	106222	53231	98721	0	0
1-Undecanol	0	0	0	71714	82503	0
Nonane, 4,5-dimethyl-	0	145176	458644	0	460415	430874
Butane, 2,2-dimethyl-	0	9526	0	0	0	0
(S)-(+)-5-Methyl-1-heptanol	0	45750	38949	0	0	0
(SS)- or (RR)-2,3-hexanediol	0	5634	6599	0	0	0
2-Methylheptanoic acid	0	19725	0	0	0	0
Cyclobutanecarboxylic acid, 1-cyclopentylethyl ester	0	15880	0	0	0	0
Butanoic acid, methyl ester	0	10909	0	0	0	0
Phthalic acid, cyclobutyl tridecyl ester	0	0	7536	0	0	0
2-Methoxybenzoic acid, 2,3-dichlorophenyl ester	0	0	2065	0	0	0
1,3-Dioxolane, 2-(1-bromoethyl)-	0	0	7666	0	0	0

1,1'-Bicyclopropyl, 2,2,2',2'-tetramethyl-	0	0	18756	0	0	0
9-Octadecenoic acid (Z)-, methyl ester	0	0	51755	0	0	0
Butane, 2-bromo-2-methyl-	0	0	4821	0	0	0
Tridecanoic acid, methyl ester	0	0	25987	0	54120	35338
Benzoic acid, 4-methyl-, phenyl ester	0	0	11695	0	0	0
Trehalose	0	0	0	0	1475711	0
Trichloroacetic acid, undecyl ester	0	0	0	0	147878	0
Tryptamine	0	0	0	0	42685	0
1-Dodecanol	0	0	0	0	82503	0
Bicyclo[5.3.0]decane	0	0	0	0	64316	0
9,12,15-Octadecatrienoic acid, 2,3-dihydroxypropyl ester, (Z,Z,Z)-	0	0	0	0	107307	0
Cyclohexanol, 5-methyl-2-(1-methylethyl)-, [1S-(1.alpha.,2.beta.,5.beta.)]-	0	0	0	0	49854	0
Tetradecanoic acid, 12-methyl-, methyl ester, (S)-	0	0	0	0	38564	0
Hexanedioic acid, mono(2-ethylhexyl)ester	0	0	0	0	68447	0
Tridecanedioic acid, dimethyl ester	0	0	0	0	42560	0
Diisooctyl phthalate	0	0	0	0	289982	59630
Nonane, 1-iodo-	0	0	0	0	0	33278
Silane, [(1,1-dimethyl-2-propenyl)oxy]dimethyl-	0	0	0	0	0	14058
4,4-Dimethyl-2-pentanol, trimethylsilyl ether	0	0	0	0	0	14596
4-Dodecanol	0	0	0	0	0	23205
17-Octadecynoic acid, methyl ester	0	0	0	0	0	36054
Ethyl aminomethylformimidate	0	0	0	0	0	19375
Hexanedioic acid, bis(2-ethylhexyl) ester	0	0	0	0	0	15297
4H-Pyran-4-one	0	0	0	0	0	14302
Acetic acid, cesium salt	0	0	0	0	0	26807
3,4-Dimethyl-1-dimethyl(trimethylsilylmethyl) silyloxycyclohexane	0	0	0	0	0	10199

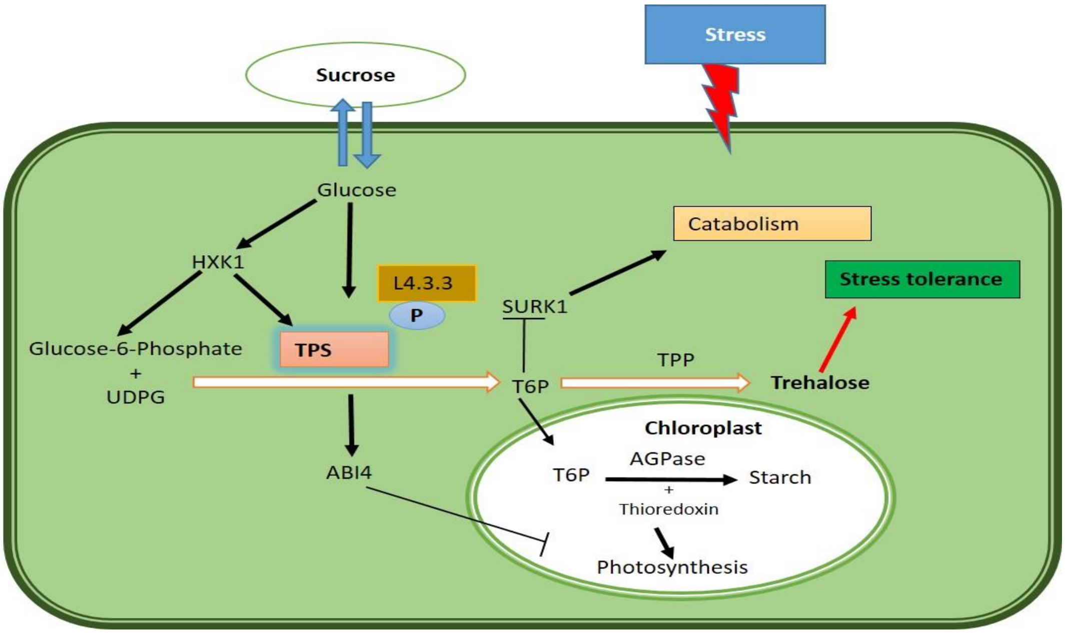

Figure S1: Trehalose pathway role in eukaryotes. Trehalose-6-phosphate (T6P) control sugar metabolism and plant development. Trehalose and glucose are also responsible for many signaling and regulatory pathways and integrate external cues to adapt cells to abiotic stress, growth and development The diagram is adapted from [103]. Metabolites detected in shoots

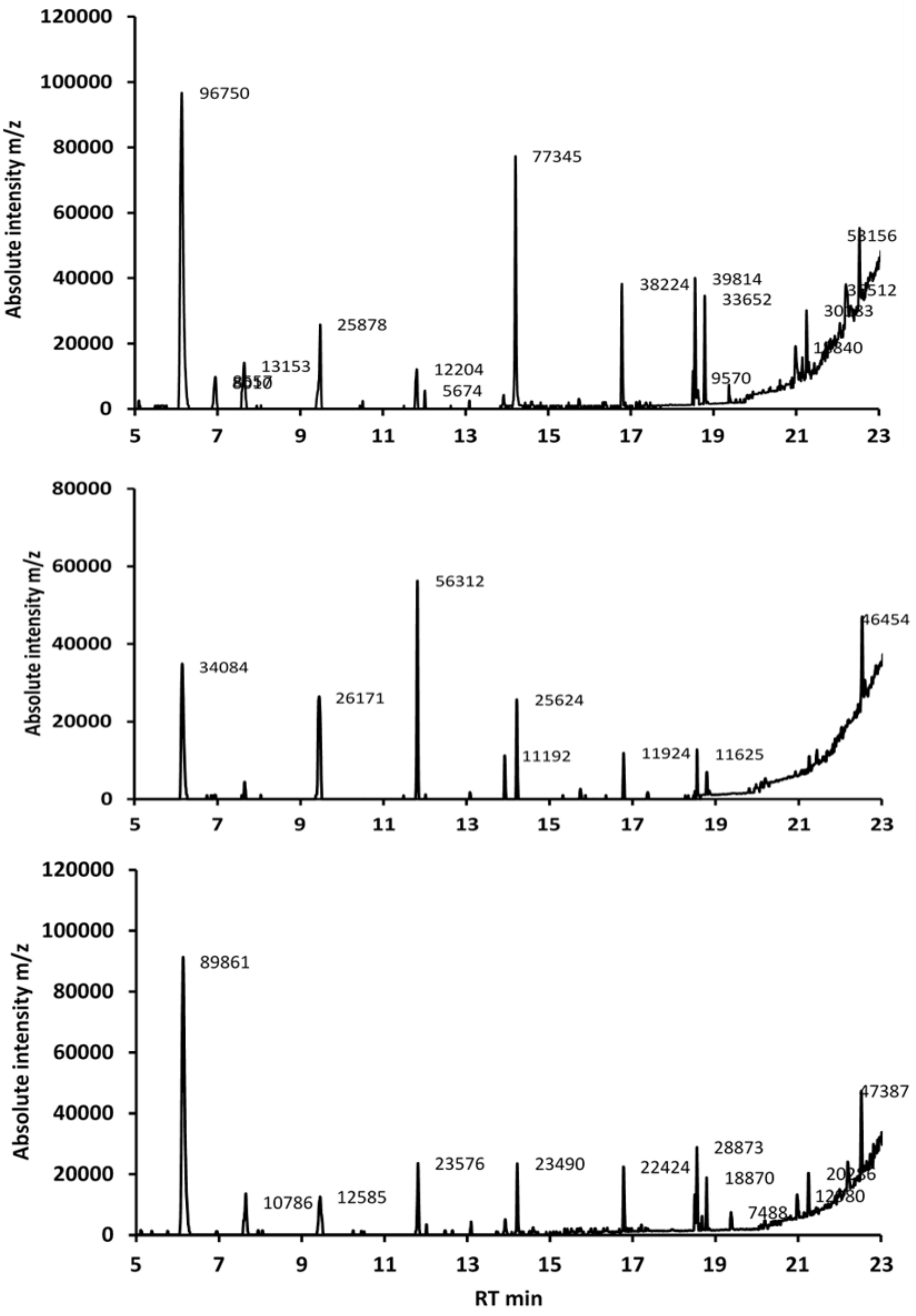

Figure S2. GC-MS chromatography intensity readings of cv. 'BARI-41'. Control (top), LiCl 20mM (middle), Mannitol 150 mM (bottom)

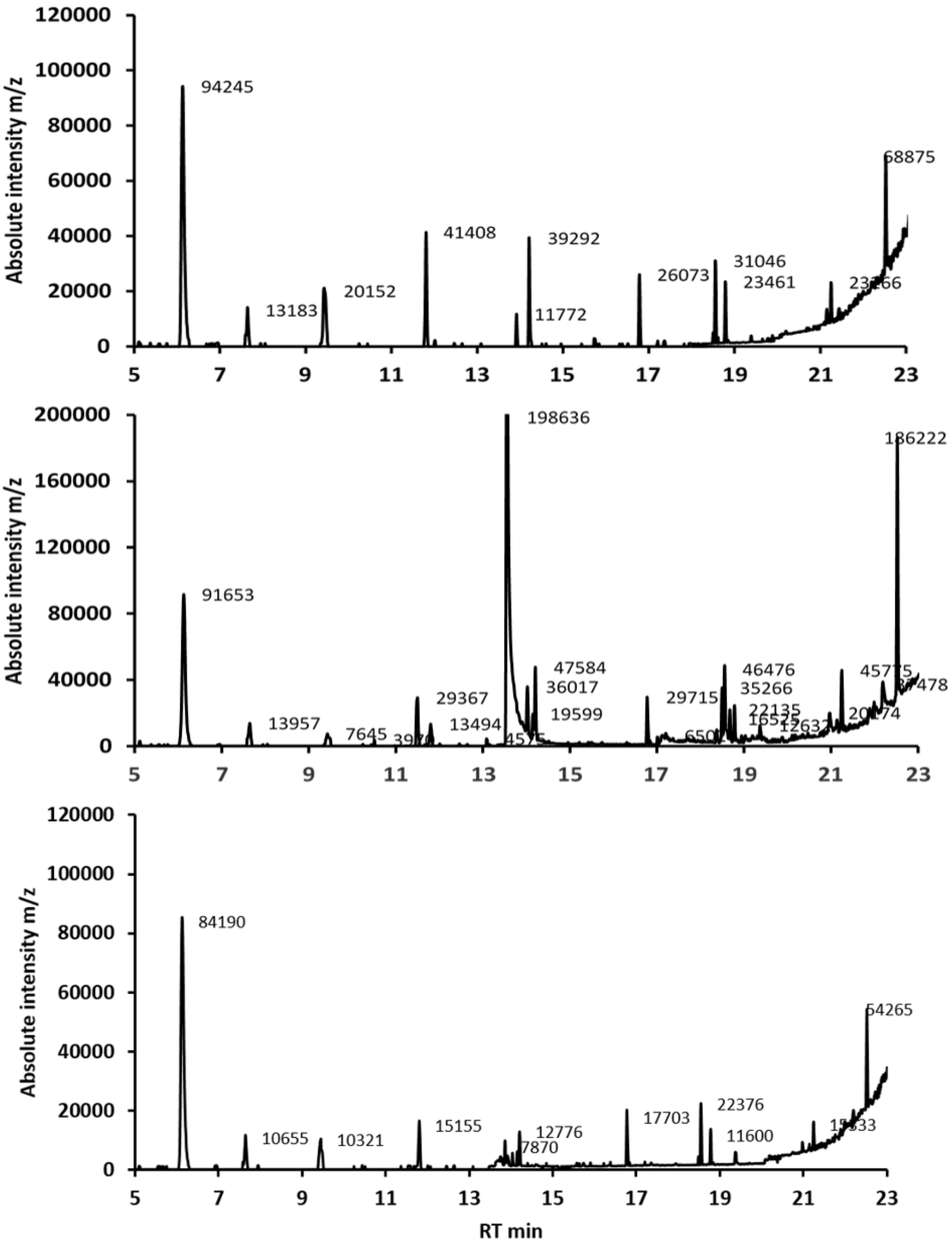

Figure S3. GC-MS chromatography absolute intensity readings of cv. 'Spunta'. Control (top), LiCl 20mM (middle), Mannitol 150 mM (bottom)
